# Single-cell spatial transcriptomics in cardiovascular development, disease, and medicine

**DOI:** 10.1016/j.gendis.2023.101163

**Published:** 2023-11-14

**Authors:** Songjie Han, Qianqian Xu, Yawen Du, Chuwei Tang, Herong Cui, Xiaofeng Xia, Rui Zheng, Yang Sun, Hongcai Shang

**Affiliations:** a Key Laboratory of Chinese Internal Medicine of Ministry of Education, Dongzhimen Hospital, Beijing University of Chinese Medicine, Beijing 100700, China; b School of Life Sciences, Beijing University of Chinese Medicine, Beijing 102488, China

**Keywords:** Cardiovascular disease, Precision medicine, Single-cell spatial transcriptomics, Single-cell transcriptome, Spatial transcriptome, Transcriptomics

## Abstract

Cardiovascular diseases (CVDs) impose a significant burden worldwide. Despite the elucidation of the etiology and underlying molecular mechanisms of CVDs by numerous studies and recent discovery of effective drugs, their morbidity, disability, and mortality are still high. Therefore, precise risk stratification and effective targeted therapies for CVDs are warranted. Recent improvements in single-cell RNA sequencing and spatial transcriptomics have improved our understanding of the mechanisms and cells involved in cardiovascular phylogeny and CVDs. Single-cell RNA sequencing can facilitate the study of the human heart at remarkably high resolution and cellular and molecular heterogeneity. However, this technique does not provide spatial information, which is essential for understanding homeostasis and disease. Spatial transcriptomics can elucidate intracellular interactions, transcription factor distribution, cell spatial localization, and molecular profiles of mRNA and identify cell populations causing the disease and their underlying mechanisms, including cell crosstalk. Herein, we introduce the main methods of RNA-seq and spatial transcriptomics analysis and highlight the latest advances in cardiovascular research. We conclude that single-cell RNA sequencing interprets disease progression in multiple dimensions, levels, perspectives, and dynamics by combining spatial and temporal characterization of the clinical phenome with multidisciplinary techniques such as spatial transcriptomics. This aligns with the dynamic evolution of CVDs (*e.g.*, “angina–myocardial infarction–heart failure” in coronary artery disease). The study of pathways for disease onset and mechanisms (*e.g.*, age, sex, comorbidities) in different patient subgroups should improve disease diagnosis and risk stratification. This can facilitate precise individualized treatment of CVDs.

## Introduction

In the last 50 years, mortality due to cardiovascular diseases (CVDs) has reduced owing to the rapid advancements in public health, clinical cardiology, and science and technology.^1^ Nevertheless, the global prevalence of CVDs is still high. Owing to their high morbidity and mortality, CVDs are among the most important diseases affecting human health, especially considering that they are a primary cause of death. CVDs account for approximately 30% of deaths globally, surpassing other diseases including cancer. The estimated cost incurred for the treatment of CVDs is predicted to increase to $10.44 trillion by 2030.^2^ CVDs are also associated with high rates of sudden death and disability, and their onset irreversibly affects the patients. Even with the most advanced and sophisticated treatments, more than 50% of accidental CVD survivors may be unable to care for themselves.^3^ CVDs also have a high recurrence rate,^4^ which ranges from 8.7% to 29.6%. CVDs can recur many times, and the severity of CVDs increases with each recurrence.^5^ In addition, the clinical translation of therapeutic strategies targeting widely functional signaling pathways involved in inflammation and fibrosis has not been successful. As CVDs damage the heart, the heart's function of pumping blood is weakened in their later stages. Long-term insufficiency of blood pumping may lead to hypoxia, blood stasis, and conditions that affect the functioning of other organs. Therefore, the development of novel biomarkers and precision medicine tools for personalized risk stratification and treatment allocation in future clinical trials and new high-resolution technologies to identify highly specific therapeutic targets are warranted.^6^ For this purpose, it is critical to obtain a better understanding of molecular mechanisms and disease subclasses. The occurrence and progression of CVDs involve complex molecular mechanisms of various cell types, subtypes, and states. Studies of single-cell multiomics transcription techniques can provide comprehensive information regarding cell type, molecular heterogeneity, and spatial distribution.

Recent improvements in histological technologies have greatly enhanced our understanding of the mechanisms and cells involved in CVDs. Single-cell RNA sequencing (scRNA-seq) can identify disease- and cell-type-specific gene regulatory networks or new potential biomarkers and estimate cell–cell and receptor–ligand interactions. It also allows cell trajectory analysis based on the transcriptome of individual cells, which helps in elucidating the evolution of cell states during the development and differentiation process of progenitor or stem cells.^7^ Nonetheless, scRNA-seq does not offer the spatial resolution required to obtain a reliable understanding of cell–cell interactions, cellular microenvironments, and spatial differences in cells and molecules in CVDs.^8^ In this context, spatial transcriptomics (ST) can facilitate three-dimensional, nonhomogeneous, and precise analysis. ST adds spatial information to scRNA-seq data, deepening our understanding of niches of local tissues that constitute the cellular microenvironment and promote cell–cell interactions, in addition to clarifying the genes and proteins that depend on this spatial environment in three-dimensional tissue structures. It can thus precisely elucidate the molecular mechanisms of cardiac injury and remodeling.^9^^,^^10^ Based on this, this review provides a comprehensive overview of single-cell and ST technologies and recent research on these topics in the field of CVD research. This work may provide a basis for the development and elucidation of the potential application directions and future research priorities of this technology in disease.

## Single-cell spatial transcriptomics

### Single-cell transcriptome sequencing

Humans contain approximately 3.72 × 10^13^ cells of various types, which are the operative basis of organisms and biological processes. These cells form harmonious microenvironments to maintain appropriate organ functions and cellular homeostasis.^11^ Although all cells in the human body share the same genetic material, their transcriptomes reflect the unique activities in only a subset of genes. Genetic diversity leads to cellular heterogeneity, which is phenotypically altered by perturbations of genetic or environmental regulation and reflected at multiple molecular levels.^11^ The transcriptome links cellular phenotype and genotype and interprets various biological phenomena through molecular composition and regulators. Therefore, to probe cellular identity, state, function, and response, it is practical to profile the gene expression activity in cells.^12^

scRNA-seq is a new technology for high-throughput sequencing of cellular transcriptomes at the single-cell level. Since Tang et al^11^ made the first breakthrough in single-cell transcriptomics approaches in 2009, single-cell transcriptomics has revolutionized. Its principle involves the efficient amplification of trace amounts of RNA or DNA from individual cells without requiring selective cell purification and the performance of high-throughput sequencing.^11^ With scRNA-seq, the single-cell transcriptomes of millions of cells can be analyzed in a single study. Moreover, single-cell technologies measure different types of RNA transcripts or epigenetic modifications. scRNA-seq, serving as a molecular microscope, can comprehensively delineate transcriptome diversity, parse cell-to-cell heterogeneity, elucidate relationships between cells, and identify rare cell populations.

### Main methods

Many modified and improved scRNA-seq technologies have been developed to introduce essential modifications and improvements in sample collection, single-cell capture, barcoded reverse transcription, cDNA amplification, library preparation, sequencing, and streamlined bioinformatic analysis. The main procedures of scRNA-seq include single-cell isolation and capture, cell lysis, reverse transcription (conversion of their RNA into cDNA), PCR amplification, high-throughput sequencing, and data analysis.^13^

#### Single-cell isolation^14^, ^15^, ^16^, ^17^

Single-cell isolation involves the isolation of single cells from heterogeneous cell populations or the capture of high-quality single cells from tissues. Isolation of single cells is the first and crucial limiting step in scRNA-seq. Cells must be liberated from the extracellular matrix and cell–cell adhesion (except for circulating cells), which is often achieved by enzymatic treatment. Cell isolation can be accomplished by isolating whole cells, cell-specific nuclei, or cell-specific organelles and separating the desired cells expressing specific marker proteins. The choice of single-cell isolation method will vary depending on the organism, tissue, or cell properties. There are currently three main single-cell isolation techniques: limiting dilution, fluorescence-activated cell sorting, and magnetic-activated cell sorting.

#### Single-cell capture^18^

Single-cell capture involves the extraction of precise genetic and biochemical information and elucidation of unique activity observed in only a subset of genes and their molecular mechanisms. The used capture technology determines how cells are screened, what supplementary information to obtain, and what data are yielded. The three most commonly used methods are based on microwell, microfluidic, and droplet methods. Microwell-based capture techniques employ cell pipettes or laser capture to separate cells and place them in microfluidic wells. One of the advantages of this approach is that it can be combined with fluorescence-activated cell sorting to achieve surface marker-based cell selectivity. Therefore, this technique can be helpful for studies that aim to isolate specific cell populations. Another advantage of this technique is that cells can be photographed to help determine whether damaged or duplicated cells are present in the wells. However, it has the disadvantage of low throughput, requiring laborious operations in each cell. Droplet-based methods segregate cells into individual aqueous compartments in a lipid suspension. Cells are lysed within droplets, and mRNA molecules are reverse-transcribed and tagged with oligonucleotide barcodes uniquely identifying the cell. In addition, the randomness of cell barcodes is free of cell size or morphology. Droplet-based scRNA-seq is an efficient technique as it requires a small reaction volume and can rapidly compartmentalize thousands of single cells per minute by rapid droplet encapsulation.

#### Cell lysis ADDIN^11^

Cell lysis involves the lysis of cells to obtain genetic material. The lysis separation strategy can be used to analyze cell functions in parallel. This strategy can detect mRNA in the cytoplasm and genomic DNA in the nucleus. The currently used methods can be divided into physical, chemical, and biological enzyme-based methods.

#### Reverse transcription and PCR amplification^13^^,^^18^

Reverse transcription involves the isolation of RNA and its conversion into cDNA for further analysis. Amplification of DNA and RN contents of a single cell is required when their content is insufficient for sequencing. PCR amplification involves the conversion of RNA into first-strand cDNA, and the resulting cDNA is amplified by either PCR or *in vitro* transcription. There are currently two PCR amplification strategies available, including SMART technology. The strategy connects the 5′ ends of cDNA with either poly(A) or poly(C) to build standard adaptors in the PCR reaction.

#### High-throughput sequencing^19^

Once a single cell or monocyte has generated a single-cell barcoded cDNA, the cDNA can be sequenced. A remarkable chain of the PCR product is reverse complementary to a particular molecule. It is connected to the single-stranded molecule by DNA ligase. Finally, a single-stranded circular DNA library is obtained. Various scRNA-seq technologies and experimental protocols are available.

#### Data analysis^11^^,^^20^

Analysis of scRNA-seq data is of paramount importance to broaden the application of this technology in life and clinical sciences. An increasing number of tools are now available for single-cell transcriptome analysis, and approximately 1000 bioinformatics tools are currently available. This illustrates the importance of analytical methods in this field.

### The application of single-cell transcriptome sequencing

With the development of scRNA-seq technologies and methods, single-cell transcriptomics can be applied to model organisms and used to create reference maps of healthy human tissues, organs, and systems at single-cell resolution. Moreover, the Human Cell Atlas global consortium has used high-throughput technologies at single-cell resolution to map cells in human tissue (https://www.humancellatlas.org/), including the immune system, central nervous system, epithelial tissue, fetal cells, and cancer cells. Analyzing healthy and diseased tissues and genetic variations between individuals using scRNA-seq is critical to understanding disease mechanisms. Single-cell RNA expression profiling is rapidly becoming an irreplaceable method in various fields of research, including humans, animals, and plants.

### Biological applications^21^

#### Identification of cell types

The most important application of single-cell mRNA sequencing is to identify cell types in a complex mixture. Unbiased screening of randomly sampled cells from a mixture, such as those from an organ, can reveal the cellular composition of a sample. Therefore, a cell's transcriptome is called its fingerprint, which reveals its identity.

#### Identification of marker genes

Once the cell type is identified, data on specific marker genes can be mined, allowing efficient characterization and purification of the desired cell type, with the help of cell surface markers or fluorescent reporter genes. The discovery of marker genes requires the identification of differentially expressed genes between the cell type of interest and the remaining cells.

#### Inference of differentiation dynamics

The use of single-cell transcriptomics to identify cell type can lead to unraveling of differentiation pathways. The pseudo-chronological order of the single-cell transcriptome can be inferred by analyzing samples containing all differentiation stages of a particular cell lineage. Continuous temporal changes in gene expression accompany differentiation, and the ordering of similarity of the single-cell transcriptome reflects the continuity of these changes, resulting in a spurious temporal order of the single-cell transcriptome.

#### Measuring gene expression noise

Another application of single-cell mRNA sequencing is the investigation of biological gene expression variability or gene expression noise in a population of cells. Single-cell mRNA sequencing can infer biological noise and investigate transcriptional parameters in a cell population of interest on the whole genome level.

#### Investigating allelic expression

Single-cell sequencing offers the possibility to study allelic expression on the genome-wide level.

### Applications in plants^22^

After successfully validating scRNA-seq technology in *Arabidopsis* roots, studies have increasingly applied this technology to study other parts of *Arabidopsis* and other plant species, such as rice leaves and roots, tomatoes, and maize. The Plant Cell Atlas Consortium was established in 2019 to gather more information on various plant cell types, and their nucleic acids, proteins, and metabolites. Various web-based graphical information on plant scRNA-seq data can be found at https://www.zmbp-resources.uni-tuebingen.de/timmermans/plant-single-cell-browser.

### Application in fundamental life science investigations

With the development of technology and applications, it is expected that an increasing amount of scRNA-seq data will be generated and integrated into a publicly accessible database. This should deepen our understanding of gene and cell functions in health and diseases. scRNA-seq has become a powerful tool for analyzing, identifying, classifying, and discovering new or rare cell types and subtypes from diverse human organs and tissues. It has significantly contributed to progress in the fields of immunology, diabetes, microbiology, cancer biology, vascular biology, neurobiology, and clinical diagnosis, among many other fields. This technology has also been demonstrated to be a powerful tool for understanding infectious diseases.

#### In tumors

Understanding the tumor microenvironment with primary immunity and stromal infiltration is a prerequisite for understanding tumorigenesis; the evolutionary origin of cells; and tumor progression, metastasis, and treatment.^23^, ^24^, ^25^ scRNA-seq analysis can distinguish functional healthy cells from cancer cells at different stages of tumor development. This can lead to a more precise prognosis and diagnosis, as this will facilitate the identification and determination of sensitivity to different drugs and development of the highly effective cancer treatment strategies. To understand the evolutionary process of tumor formation and the acquisition of traits such as resistance to chemotherapy and drugs, scRNA-SEq can be used to investigate tumor heterogeneity. scRNA-seq can provide meaningful insights into the minor treatment-resistant cell populations inside complex tumors, which can be used to select appropriate therapies based on tumor type and administer appropriate treatment to all patients. This approach can be used for conditions such as melanoma, liver cancer, hepatocellular carcinoma, glioblastoma, breast cancer, prostate cancer, lung adenocarcinoma, head and neck squamous cell carcinoma, nasopharyngeal carcinoma, head and neck cancer, pancreatic cancer, and acute myeloid leukemia.^26^, ^27^, ^28^, ^29^, ^30^, ^31^, ^32^, ^33^, ^34^, ^35^, ^36^

#### In diabetes^37^^,^^38^

An essential application of scRNA-seq technology is to obtain a better understanding of β-cell development and pathology in diabetes. The cure for type 1 diabetes lies in the restoration of β cells. Therefore, scRNA-seq studies of the developing pancreas were performed in a mouse model. The following findings were made: (ii) scRNA-seq characterized the generation, expansion, and maturation mechanism of pancreatic β and α cells during pancreatic development. Moreover, the proliferation rates of β and α cells peaked at different developmental stages and were enriched in different developmental regulatory pathways for maturation. (ii) The proliferation rates of β and α cells peaked at different developmental stages and were enriched in different developmental regulatory pathways for maturation. (iii) scRNA-seq study confirmed that there is intraspecific differential expression of multiple genes in mouse and human β and α cells.

#### In infectious diseases

scRNA-seq was performed on peripheral blood^39^, ^40^, ^41^, ^42^ to identify differences in the composition of critical immune cells among patients with moderate or severe COVID-19, convalescent patients, and controls. The results showed that COVID-19 induced unique immune cell signaling compared with the findings in healthy controls, particularly during the early recovery phase.

### Spatial transcriptomics

The team of the Swedish scientist Professor Joakim Lundeberg first proposed the concept of ST in 2016. This technique was named Technique of the Year by Nature in 2020, highlighting the importance of this technology. ST technology can preserve spatial tissue location and provide transcriptome information without the need to prepare a suspension. The most significant difference between ST and single-cell transcriptomics is the loss of the tissue environment and spatial information of the cell while preparing a single-cell suspension for single-cell transcriptomics.^33^

In addition, ST plays an essential role in making connections and linking corresponding information (*e.g.*, cell–cell interactions, transcription factor distribution, cellular spatial localization, and mRNA expression) between tissue slices and molecular maps via artificial intelligence, computer programming, and visualization techniques.^43^ ST technologies visualize profiles of RNA molecules in identified tissue regions, including technologies based on micro-dissected gene expression, *in situ* hybridization (ISH), *in situ* capturing, and *in situ* sequencing (ISS) technologies. Although ST is highly variable in terms of the number of genes that can be explored and the size of tissue that can be detected, it allows transcriptome-level measurements across tissue regions and visual identification of RNA molecular profiles in tissue regions.^44^

### Main methods

ST technologies are primarily categorized as (i) next-generation sequencing-based approaches, which encode positional information onto transcripts before next-generation sequencing, and (ii) imaging-based approaches, comprising ISS-based methods, wherein transcripts are amplified and sequenced in the tissue, and ISH-based methods, wherein imaging probes are sequentially hybridized in the tissue.

#### Next-generation sequencing-based approaches

In 2016, Stahl et al^45^ reported the use of the first next-generation sequencing-based method for ST that enabled transcriptome isolation from tissue sections. Next-generation sequencing-based approaches stem from the conceptual innovations of scRNA-seq methodologies and can capture entire transcriptomes from tissue sections.^46^ The core innovation is the capture of polyadenylated RNA on spatially barcoded microarray slides before performing reverse transcription, ensuring that each transcript can be mapped back to its original point using a unique positional molecular barcode. As in all next-generation sequencing-based methods, the spatially barcoded RNAs are collected and processed for sequencing.^47^ The barcode of each read is used to map the spatial position. Conversely, the rest of the sequencing read is mapped to the genome to identify the transcript of origin, collectively generating a gene expression matrix.

#### Imaging-based approaches

Two main imaging-based approaches in ST include ISS- and ISH-based methods. ISS-based methods directly decode the sequences of transcripts within the tissue.^12^ ISH-based methods are based on ISH technologies wherein a target sequence is detected by hybridization of a complementary fluorescent probe.

### ISH-based methods

ISH hybridizes a nucleotide probe with different fluorescent stains to RNA, using one color for each hybridization. Eight rounds of hybridization are sufficient to resolve all genes in the human genome. ISH with trackable labels visualizes the gene expression of specific targets directly in the original microenvironment, including single-molecule fluorescence ISH (FISH), sequential FISH, multiplexed error-robust FISH, improved continuous FISH, cyclic single-molecule FISH, and RNAscope. Labeled nucleic acid probes determine the spatial location and abundance of cellular DNA and RNA in tissues.

a) Single-molecule FISH utilizes multiple short oligonucleotide probes (approximately 20 bp) to target different regions in the same mRNA transcript. It is a highly sensitive method that offers high-resolution subcellular spatialization. However, the generated signal is weak because each oligonucleotide is coupled to only one fluorophore. This technique warrants the use of a high number of single-molecule FISH probes to ensure sufficient cycles of hybridization, which eventually increases the test time and cost.^48^
b) Sequential FISH, a multiplexed variant of single-molecule FISH, allows repeated detection of individual transcripts by successive hybridization rounds, imaging, and probe stripping.^44^ Unlike single-molecule FISH, it has a signal amplification step that increases the signal-to-noise ratio by 20 times. However, the coverage of the whole organism by sequential FISH staining is highly dependent on the efficient exchange of macromolecules in dense tissues.
c) Multiplexed error-robust FISH is a single-molecule imaging method that combines labeling and continuous imaging to improve the quality and quantity of high-throughput detection. It reduces errors in single-molecule labeling and binary barcode detection by determining the copy number and spatial localization of massive RNA in a single cell. As this technique considers the copy number variation and spatial distribution of genes to identify spatial patterns of gene and genome coregulation, the distance between RNA targets is increased, thereby facilitating the detection of more RNA and reducing spectral overlap.^49^
d) Improved continuous FISH can read and illustrate 10,000 target mRNAs in cells multiple times without needing a super-resolution microscope while localizing. It can detect the cell type and spatial distribution in the tissue.^50^
e) Cyclic single-molecule FISH is a non-barcode technique wherein the number of targets is set to the number of hybridization rounds, and each round of hybridization includes a probe set of a certain number of genes. After each round of hybridization, an image is obtained and then the probe is removed for the next round of hybridization.^51^ Although cyclic single-molecule FISH has a lower reusability than other single-molecule FISH techniques. Moreover, it limits the number of marker genes and transcript length that can be analyzed, although it can effectively analyze significant tissue regions.
f) In 2011, Advanced Cell Diagnostics launched a commercially available RNAscope chip. The probe design is at the heart of this assay, where two adjacent “Z-probes” bind to the RNA transcript of interest to form the desired binding site, which allows amplified molecule hybridization. The maximum number of simultaneous detection RNA targets was increased to 12 in July 2019. The low-magnification RNAscope is combined with an imaging plasmid cytometer to hybridize DNA trees with metal tags instead of fluorophores. Subsequently, metal-bound antibodies are added to the same tissue section and plasmacyte levels are measured to determine metal abundance, allowing simultaneous evaluation of RNA and proteins. RNAscope is an essential technique in developing single-cell ST analysis, as it rebuilds images and locations from biomolecular colocalization data through chemical reactions to obtain precise genetic information at high spatial resolution. It maximizes the ability to detect RNA and protein targets simultaneously.

### ISS-based methods

FReverse transcription of RNA based on rolling amplification and sequencing for detecting the RNA isotype, the transcriptomic distribution, and the cell typing of the tissue sections. The unique feature of ISS-based methods is probe amplification and the used integrated barcoding system, which can be decoded across sequential rounds of probing, imaging, and stripping. To meet the requirements of studying the spatial mapping of larger gene panels in whole tissue sections, ISS method performs a second iteration using hybridization sequence chemistry to define the position of the mRNA molecule within the tissue (hybridization-based ISS). Moreover, the hybridization-based ISS method employs probe amplification and an integrated barcode system that decodes in the successive probe, imaging, and stripping rounds, allowing more powerful molecular detection, enhancing its ability to resolve gene expression *in situ* spatially, and supporting adaptation to larger gene panels.^52^

a) Laser capture microdissection: Laser capture microdissection coupled with RNA-seq separates individual cells using laser capture microscopy and measures the desired cells for capture with scRNA-seq, with a specific spatial location of the tissue specimen. Captured cells can be subjected to various analytical procedures, such as RNA-seq, which shows high coverage and can detect almost all genes expressed in the region of interest of the sample. However, the tedious experimental process and low throughput limit its emergence as a widely used ST technique.^12^
b) Microarray technology: This technique involves the arrangement of high-density nucleic acids on carrier surface in a specific order, and a specific reaction of biomolecules is used to detect biological samples. Recently, hybrid and integrated methods have achieved good results in the feature selection of microarray data. Recently, a feature selection algorithm called a nested genetic algorithm has been proposed. This method combines a *t*-test with two nested genetic algorithms, where one test analyzes microarray data and the other processes DNA data. In addition, a two-stage classification model based on feature selection and differential representation paradigm is proposed. In the first stage, the ReliefF algorithm can generate the best gene subset; subsequently, different spaces formed by the selected genes are used to construct the classifier in the second stage.

### The application of spatial transcriptomics

Since ST techniques provide unbiased images of the spatial composition. Therefore, ST was widely used to analyze the molecular spatial structure of tissues and create an atlas of biomolecules in clinical and biological research. ST has accelerated the ability to elucidate the inner workings of discrete cellular subpopulations in various environments and organ systems. ST is widely used in normal development, physiology, and diseases.

### Spatial transcriptomics in the nervous system

ST was widely used to analyze the molecular spatial structure of tissues and create an atlas of biomolecules in clinical and biological research. It has provided new insights that deepen our understanding of individual neuron types, locations, dendritic structures, axonal projections, and corresponding functions in the nervous system.^53^ ST-based approaches have established detailed maps of the entire mouse brain or specific regions such as the visual cortex, primary motor cortex, middle temporal gyrus, hypothalamic pre-optic region, hippocampus, and cerebellum.^43^ The spatial organization of adult mouse brain regions includes the molecular code used to map and target discrete neuroanatomical domains. The annotation of spatial genes in different brain regions provides new insights that help us to understand neuronal structure, neural connectivity, interneuronal projection, and presynaptic and glial interactions. The ad-related genes were identified from the ST profiles of the mouse sections, including the spatially dysregulated genes related to the stress response and mitochondrial dysfunction.^53^ Unbiased recognition and interpretation of spatial features and molecular profiles using ST could explain the structure–function relationships of circuits and behavior, and assist in the systematic classification of the adult mouse brain.

### Spatial transcriptomics in pathology

ST technology plays a role in pathology, testing, immunity, and other fields. For example, the widely used ISH technology can be applied to visually analyze the different times and spaces of hybridization in different species.^33^ It is an essential means to evaluate chromosomal abnormalities and identify different cells.

For example, in the experiments revealed by the Immunity Atlas of Interstitial Cystitis, the enrichment of immune cells in the urothelium of interstitial cystitis was proven by spatial transcriptional techniques. In contrast, the mapping of immune subsets revealed the differences in the distribution of immune cells between interstitial cystitis and control bladder tissues, providing new ideas for new perspectives on disease pathology.^12^

ST enables high-resolution assessment of spatial gene expression across tissue sections, overcoming the limitations of tissue dissociation. Therefore, the generation of spatial transcriptomic data on liver sections, combined with the knowledge of liver partition, enables the spatial annotation of structures comprising small mixtures of cells in the liver microenvironment (leaflet) and liver environment (tissue section).^33^

Spatial transcriptomes can be used to explore the cellular microenvironment. The combination of Slide-seq and targeted *in situ* RNA-seq revealed significant differences in the cellular composition of the spermatogenic microenvironment in mouse and human testis. A comparison of the spatial profile of testis production in wild-type and diabetic mice revealed the disruption of the spatial cellular organization of the seminiferous tubules as a potential mechanism of diabetes-induced male infertility.^12^

### Spatial transcriptomics in diseases

Beyond normal development and physiology, ST has advantages in studying tissue disorders in diseases. Most significantly, ST makes it possible to identify the mechanisms involved in cancer. ST has been used to investigate its relationship with cancer cells in different states. It enables the study of the molecular features across the boundaries between cancerous and normal tissues, such as an immunomodulatory cancer cell status found in cutaneous squamous cell carcinoma.^11^ It has also led to new potential insights into the mechanisms of tissue dysregulation in neurodegenerative diseases, including Alzheimer's disease and amyotrophic lateral sclerosis, along with new explanations of infection and inflammatory processes, such as in leprosy, influenza, and sepsis. Beyond this, ST has been applied to study rheumatic diseases, including rheumatoid and spinal arthritis.^33^

### Single-cell spatial transcriptomics

**Figure 1:**
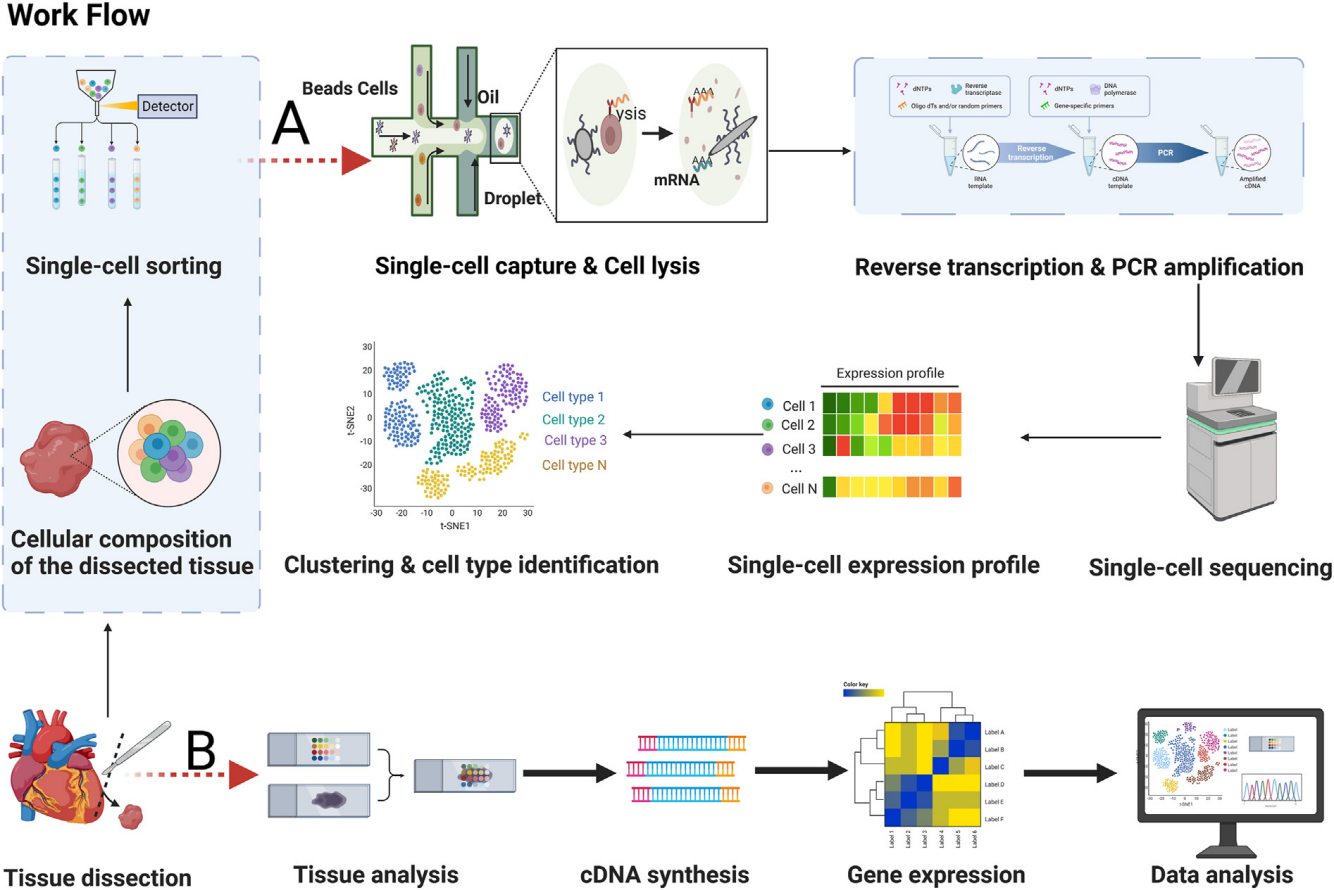
Single cell spatial transcriptomic sequencing process. **(A)** Workflow of single-cell RNA sequencing. The general experimental workflow of single-cell RNA-sequencing begins with dissociation of the organ or tissue of interest to live single cells, which requires a fine-tuned digestion protocol that maximizes cell number and cell quality while minimizing the duration of digestion and cell death. Cultured cells are likewise detached and prepared as single cells. Prepared cells are then captured by various methods of single-cell capture. Reverse transcription of single-cell RNA is performed, followed by PCR amplification and library preparation of the resulting cDNA. Next-generation sequencing is subsequently performed to generate the readouts, which are aligned to a reference genome, processed for quality control and analyzed by the user. **(B)** Workflow of spatial transcriptomics. The entire transcriptome was captured from the tissue sections. Polyadenylated RNA was captured on the spatial barcode microarray slides prior to reverse transcription, and the spatial barcode RNA was collected and processed for sequencing. Barcodes for each read were used to map the spatial locations, while the rest of the sequencing reads were mapped to the genome to identify the transcripts of the origin, to jointly generate a gene expression matrix.

scRNA-seq can perform an unbiased screening of a random sample of cells in a given organ and reveal the subpopulation composition of cells within a fixed organ. However, in the necessary tissue separation step of scRNA-seq, the isolation of individual cells disrupts their spatial localization in natural tissues and the exchange of information among them. Thus, scRNA-seq cannot capture their spatial distribution or reveal the local *in situ* acting intercellular communication network. Therefore, it is possible to induce ectopic gene expression, leading to the misrepresentation of specific cell subpopulations. Nonetheless, ST provides transcriptome expression information with its unique spatial resolution and the corresponding two-dimensional spatially organized location, elucidating the ecological sites of enrichment of different gene sets. However, the current state of ST alone does not yet provide comprehensive transcriptomic information about precisely localized single cells in the tissue. However, using a combination of two technologies can overcome these limitations (Fig. 1). ST can quantify mRNA by transcriptomics in intact tissue sections, adding spatial context to the scRNA-seq dataset and depicting the cellular microenvironment and individual cells while retaining spatial information. This is critical for understanding how cellular neighborhoods and disease pathology affect gene expression, cellular state, cellular abundance, and monitoring molecular interactions between tissue components.

## Single-cell spatial transcriptomics in cardiovascular phylogeny

### Generation of cardiovascular cell atlases

Single-cell ST techniques have been applied to identify and generate cardiovascular cell profiles, as shown in Table S1. Sixteen studies have analyzed the single-cell transcriptomics of the heart in different embryonic periods (e8.5–e21). In 2016, Li et al^54^ collected 2233 scRNA-seq samples from different stages of embryonic mouse hearts to investigate the spatial pattern gene expression characteristics of developing cardiomyocytes. Unsupervised analysis was used to identify cell types. Their study identified transcriptional markers in the left and right atria, left and right ventricles, outflow tract, navicular duct, and trabecular myocardium, demonstrating the first comprehensive assessment of transcriptional profiles of deeply sampled individual cardiac cells in the embryonic mouse heart. They also generated a comprehensive transcriptomic database. Their data resource revealed the anatomical patterns of gene expression. It enabled the prediction of the anatomical location of individual cardiomyocytes during development with >91% accuracy, providing insight into developmental perturbations in models of congenital heart defects. In 2017, Daniel et al^55^ performed scRNA-seq on more than 1200 mouse cells isolated from the original heart tube to the mature heart at seven time points. Cardiomyocytes, endothelial cells, and fibroblast-rich cells were classified using unbiased transcriptional data. This spatiotemporal transcriptome analysis of heart development revealed lineage-specific genetic programs for normal heart development and congenital heart disease, providing a powerful benchmark for assessing the maturity of stem cell-derived cardiomyocytes. The results also showed that loss of the homeobox protein Nkx2.5 impacts cardiomyocyte maturation, resulting in human heart malformations. In 2019, Asp M et al^12^ used single-cell ST to systematically analyze 3717 human heart cells at three developmental stages during the first trimester (4.5–5, 6.5, and 9 weeks after gestation). By exploiting the spatial exploration capacity of ST, the deconvolution ability of scRNA-seq, and the subcellular targeting accuracy of ISS, they revealed the heterogeneity of cells, restored the spatial location of cells, and generated a final three-dimensional transcription map. In 2020, Feng et al used ADDIN^11^ to simultaneously analyze more than 9000 cells from eight samples of mouse embryonic hearts using drop-based scRNA-Seq and individual cells from different genetic backgrounds, developmental stages, or anatomical locations. In 2019, Cui et al^56^ used scRNA-seq to analyze gene expression profiles of approximately 4000 cardiac cells from 18 human embryos. They identified four major specific cell types in the human fetal heart: cardiomyocytes, cardiac fibroblasts, endothelial cells, and valvular stromal cells. The study also found that cardiomyocytes in the atria and ventricles acquire different characteristics early in heart development, with both cardiomyocytes and fibroblasts showing progressive changes in gene expression. Most importantly, the study also compared gene expression profiles between humans and mice, showing that cardiomyocytes in E10.5 embryonic mice and 7-week-old human infants were the best matches. E10.5 mouse endothelial cells were most similar to 6-week-old human endothelial cells, whereas 5-week-old human fibroblasts were most similar to E9.5 mouse cardiomyocytes. These findings suggest that mouse and human heart development is not synchronized for specific cell types. Compared with 7-week-old human extracellular matrix genes, human ventricular cardiomyocytes expressed more extracellular matrix genes than mouse ventricular cardiomyocytes, such as COL1A1 (collagen I), COL6A3 (collagen VI), DCN, and LUM. In addition, RELN, a marker of atrial trabecular cardiomyocytes in the human heart, is not expressed in mice, and Sall4 and Refed1 are highly expressed in mouse ventricular cardiomyocytes, which may easily lead to mouse heart defects. However, these genes are not important for the human fetal heart. High expression of CITED1 in mice is associated with cardiac malformation, whereas its expression in human hearts is deficient. These findings indicate significant differences in gene expression profiles between human and mouse fetal hearts. Using scRNA-seq, Cho et al^56^ identified no difference between adult cardiomyocytes derived from pluripotent stem cells and normal human cardiomyocytes after transplantation into neonatal hearts. This lays a foundation for understanding the maturation and pathogenesis of human cardiomyocytes and can help model adult heart disease based on psc. Using scRNA-seq, Soysa et al^57^ found that Hand2 was an indicator of outflow tract cells, but not right ventricular cells, in human body weight, despite the failure of right ventricular formation in HAND2-null mice. Hand2 depletion also leads to dysregulation of retinoic acid signaling and disruption of anterior and posterior patterned cardiac progenitor cells. In addition to embryonic heart studies, cardiac fibroblasts, vascular wall cells, macrophages, cardiac endothelial cells, pericardial cells, myocardial B cells, cardiac model cells, and non-cardiac cells have been studied using the scRNA-seq technique. It has also been used to investigate the diversity of poorly defined cell species and the extensive intercellular communication network.

### Specific areas of the heart

Single-cell ST techniques were also applied to obtain transcriptional maps of specific heart regions, as shown in Table S2. Twenty-four studies were conducted involving 18 unique cardiac regions, including a sizable saphenous vein, sinus node, atrioventricular tube, coronary vessels, cardiac neural crest, ventricular septum, cardiac conduction system, cardiac parasympathetic nerve, and cardiac outflow tract, in five species (human, mouse, rat, chicken, and pig). In 2021, Liang et al^58^ used scRNA-seq technology for the first time to map the sinoatrial node cells, revealing four main cell clusters in the sinoatrial node and the core gene regulatory network controlling SAN function in mice. Moreover, in 2022, Gandhi et al^59^ revealed cardiac crown-specific transcription factors using scRNA-seq technology. Ectopic expression of the transcriptional subcircuit composed of Tgif1, Ets1, and Sox8 may enable the cardiac crest to rescue persistent trunk arteries after cardiac crest ablation in human heart defects. These findings reveal new genes involved in congenital CVDs. Elsewhere, transcriptomic analysis of the developing mouse cardiac conduction system by William et al^60^ identified clusters of cells representing each vital component of the conduction system, providing a transcriptional map of the entire conduction system. In addition, it was revealed that new subtypes of conduction cells include clinically relevant but poorly characterized “transitional cells”. This study provides a genetic map to facilitate future efforts to identify, isolate, and characterize conductive cells in developmental and disease contexts.

Liu et al^61^ performed scRNA-seq on 55,611 cells in the outflow tract of mouse hearts and revealed the molecular characteristics of six cell lineages and their subpopulations. Subsets of intermediate cells were involved in the transdifferentiation of myocardium to vascular smooth muscle cells or the transition from mesenchyme to vascular smooth muscle cells, and transcriptional regulators that may control cell transition were also identified. This provides a single-cell map of the cellular state of normal cardiac outflow tract development. The results of this study lay a foundation for further study of the etiology of cardiac outflow tract malformation at the single-cell level. Moreover, Alexia et al^62^ used scRNA-seq to analyze mouse aortas and mitral valves at postpartum P7 and P30. Cellular composition and heterogeneity in postpartum heart valve remodeling were comprehensively analyzed, and the vital cellular subsets involved in postpartum collagen production and extracellular matrix remodeling were identified. This revealed for the first time the cellular diversity of the heart valve, which opened up a new avenue for studying the molecular and cellular mechanisms of heart valve homeostasis and disease.

## Application of single-cell spatial transcriptomics in CVDs

In addition to constructing a high-quality physiological cellular atlas of the cardiovascular system, single-cell ST technology is uniquely suited for analyzing the pathogenesis and diagnosis of CVDs, with far-reaching implications for personalized medicine and targeted therapy. Studies have shown that single-cell ST has dramatically improved our understanding of the molecular mechanisms of CVDs, thereby revealing new potential diagnostic and therapeutic targets. Here, we provide an overview of recent studies on single-cell ST in CVDs and highlight their potential for target and biomarker discovery. Over 40 CVDs, such as heart failure, myocardial infarction, atherosclerosis, myocardial hypertrophy, Kawasaki disease, and hypertension, are covered here.

### Heart failure

The studies performed to date identified the constituent types of cardiomyocytes and non-cardiomyocytes during the progression of heart failure, obtained a comprehensive picture of cardiomyocyte phenotypic transformation, and determined the phenotypic transformation of cardiomyocytes, endothelial cells, and fibroblasts via animal experiments. It was revealed that a high-inflammation transcriptome in peripheral blood monocytes and T cells of patients with heart failure carrying clonal hematopoietic driver DNMT3A mutations exacerbates chronic heart failure.^63^ The results of *in vitro* experiments showed that the expression level of natriuretic peptide might be a useful diagnostic marker for heart failure.^64^ In addition, it was found that the cardiac dopamine D1 receptor can be a therapeutic target to prevent ventricular arrhythmia associated with heart failure,^65^ along with a demonstration that anti-miR-21 drugs effectively inhibit cellular remodeling.^66^ Histone deacetylation inhibitors and heat shock protein inhibitors have also been identified as potential new anti-heart failure drugs.^67^ Moreover, Ko et al identified Htra3 (high-temperature requirement A serine peptidase 3) in cardiac fibroblasts as a critical regulator of cardiomyocyte homeostasis and a regulator of cardiac fibrosis by scRNA-seq. Htra3 is vital in cardiac endocytosis and transforming growth factor-β degradation. The combined ST technique in a mouse model of heart failure (transverse aortic constriction) and heart failure caused by myocardial infarction using Htra3-knockout mice confirmed the increased expression of transforming growth factor-β and IGFBP7 (insulin-like growth factor binding protein 7) in the infarcted region of knockout mice, suggesting that IGFBP7 secreted by failing cardiomyocytes is a valuable biomarker for advanced heart failure in humans. This in turn confirmed Htra3-Ttra3's regulation of cardiac fibroblasts and IGFBP7's role as a crucial regulator of cardiac fibrosis^68^ (Table S3).

### Myocardial infarction

Post-myocardial infarction heart can be divided into three zones: infarct, border, and distal. Each zone is characterized by different cell types, cell–cell interactions, epigenomes, transcriptomes, proteomes, and metabolomes, which change over time after myocardial infarction. There are significant differences in the pathways and cells involved at different stages after myocardial infarction. Healing after myocardial infarction is also highly heterogeneous in the spatiotemporal context. The molecular mechanisms vary considerably over time in other regions of the heart. Here we present studies of single-cell ST in myocardial infarction performed to date and their significant findings. This illustrates the potential of single-cell spatial omics to obtain a deeper understanding of cell–cell interactions, cell abundance, distribution of cell subtypes and spatial states, and cellular microenvironment, and to discover further targets (Table S4).

The current study constructed a complete database that provides a transcriptional basis for neonatal heart regeneration at single-cell resolution^69^ and presented a single-cell gene expression profile of cardiac-specific resident endothelial cells and a transcriptional hierarchy supporting endogenous vascular repair after myocardial infarction.^70^^,^^71^ At the same time, local tissue-specific processes in the ischemic heart and the immunological characteristics of myocardial infarction at the single-cell level have been revealed.^72^ Studies have also identified seven prognostic markers,^73^ which revealed that B cells infiltrate the heart via the CXCL13–CXCR5 axis after myocardial infarction and contribute to local transforming growth factor-β1 production.^74^ At the same time, local tissue-specific processes in the ischemic heart and the immunological characteristics of myocardial infarction at the single-cell level have been revealed.^75^ In the first few days after myocardial infarction, endothelial cells are activated transiently,^76^ which promotes endothelial cell migration and clonal expansion to regenerate the vascular network. Sparc, which is highly expressed by cardiac Tregs, is a critical factor in protecting the heart from myocardial infarction by increasing collagen content and promoting the maturation of the infarct zone.^77^

scRNA-seq analysis revealed a crucial role for collagen triple helix repeat containing 1 as a novel regulator of myocardial infarction scar healing via cardiac fibroblasts after myocardial infarction. The urokinase fibrinogen activator receptor^78^ provides a molecular biomarker for the diagnosis and prognosis of acute myocardial infarction. At the same time, CXCR7, which is abundantly expressed in cardiomyocytes, may act as a β-blocker-biased receptor.^79^ Meanwhile, PRKAR1A and SDCBP may serve as novel biomarkers for early diagnosis,^80^ and the monocyte-associated biomarkers CUX1, CTSD, and ADD3 can be used for early prediction of post-acute myocardial infarction development of heart failure and potential therapeutic targets after heart failure caused by acute myocardial infarction.^81^ Finally, findings have shown the histologically regenerative formation of human myocardial bundles in mice with myocardial infarction after receiving cardiovascular progenitor cell transplants from the hESC lineage and providing clinical quality cells for regenerative cardiology.^82^

### Atherosclerosis

The current findings provide a transcriptome-based cellular landscape of human atherosclerotic plaques, revealing the frequency of leukocytes in 126 human plaques.^83^ A total of 14 distinct cell populations were identified, including endothelial cells, smooth muscle cells, mast cells, B cells, bone marrow cells, and T cells. Multiple cellular activation states were determined, and cellular plasticity and intercellular communication at disease sites were highlighted.^84^ It was also shown that increased plaque Tregs are characteristic of plaque regression and Treg depletion prevents plaque remodeling and contraction, which in turn impairs markers of inflammation regression.^85^ In addition, it was revealed that lipid-loaded plaque macrophages are not likely to drive lesion inflammation,^64^ whereas resident macrophages (res-like) play an unfavorable role in the atherosclerotic aorta.^86^ Concurrent studies have shown that the overexpression of CXCL3, GK, FPR1, and LST1 is an advanced recognition and intervention factor for unstable plaques, which may become targets for the prevention of atherosclerotic rupture.^87^ Furthermore, JAG1-NOTCH4 perception of perturbed flow was shown to enhance atherosclerosis susceptibility by modulating endothelial cell heterogeneity, and therapeutic targeting of this pathway may treat atherosclerosis.^88^ Finally, phenotypic and metabolic heterogeneity of endothelial cells characterizes diabetes-associated atherogenesis^89^ (Table S5).

### Other diseases

Peripheral blood mononuclear cells were characterized by scRNA-seq in human Kawasaki disease, and immune cells were disrupted at the single-cell level, constructing a single-cell landscape of innate and adaptive immune responses. Using scRNA-seq, Zhang et al^90^ comprehensively characterized vascular cells and aortic arches in mice and humans. Transient angiotensin-converting enzyme inhibition altered cardiac fibroblast subpopulations and activation levels, resulting in a reduced overall fibrotic phenotype. At the same time, aortic vascular cells associated with hypertension were found to have a favorable transcriptional landscape in which KLF10 or IL-9 in T cells may represent novel therapeutic targets for treating vascular or fibrotic disease. In addition, Wu et al^91^ explored markers of hypoplastic left heart and their molecular mechanisms using scRNA-seq and ST techniques. They found that hypoplastic left heart is genetically heterogeneous. However, many mutations are associated with sequential cellular processes that primarily drive cardiac myogenesis, with three central genes (MMP2, COL5A1, and B2M) holding promise as therapeutic targets for hypoplastic left heart. Boogerd et al^54^ explored the mechanisms of valvular heart disease using scRNA-seq by creating transmural epicardial to endocardial gene expression profiles of exposed arrhythmogenic cardiomyopathy hearts. They found that the cellular landscapes of four types of heart valves (aortic, pulmonary, mitral, and tricuspid) differ and that zinc finger and BTB structural domain-containing protein 11 may be novel drivers of cardiomyocyte loss. In addition, scRNA-seq plays a vital role in CVDs, such as vascular malformations, myocardial fibrosis, and congenital heart disease (Table S6).

## Discussion

In the last few years, with the advent of scRNA-seq and ST, transcriptomics has begun to drive discoveries across disciplines that were previously impossible with traditional bulk analysis methods. Although spatial polytomies are currently less used than scRNA-seq technology, their findings show significance in basic and clinical research far beyond the application of a single technology. Single-cell ST has excellent potential to improve our understanding of the molecular mechanisms of CVDs and thus reveal new potential diagnostic and therapeutic targets.^92^ scRNA-seq can predict cell type specificity and help select targets with limited adverse side effects. ST can also be used to add the spatial context, which helps in understanding the cellular microenvironment.^53^ It can help to localize specific locations, cell–cell interactions, and specific cardiac regions to which different targets can be targeted. For example, in the field of coronary artery disease, only the combination of scRNA-seq and ST technology can help construct dynamic three-dimensional stereoscopic single-cell transcriptional profiles in the ongoing progression from angina to myocardial infarction to heart failure, restore cellular spatial location and structure, and dynamically target specific regions for immune cell specificity. Combining phenomics and machine learning for patient-specific phenotypic tagging can identify specific gene regulatory programs in patients with different phenotypes (*e.g.*, remote regions of the infarct area, border regions, differences in the infarct area). This will provide sufficient evidence to support individualized, targeted therapy.

Owing to the significant development of multiomics technologies such as single-cell ST, we have gained new insights into the cellular microenvironment and spatial heterogeneity of CVDs. The results of this review show that the techniques of spatial polytomies have not been well disseminated. Only 3 of 22 studies related to myocardial infarction and 1 of 13 studies related to heart failure used ST. To achieve individualized and precise treatment, not only the analysis of patient cell kinetics but also the analyses of the transcriptome, metabolites, and protein expression are needed. Differences in sex, age, disease history, genetics, and living environment will also determine the patient's subtype. Therefore, the molecular regulatory mechanisms between different subtypes and targeted therapies also vary from person to person. Currently, there are no established multiomics spatial technologies due to the limited number of clinical samples, complexity of the workflow, cost of expensive assays, and limitations of the analytical methods, such as the number of detectable epitopes and specific antibody sets. However, in the future, the rapid development of emerging single-cell ST, spatial metabolomics, spatial proteomics, and spatial polytomies, as well as machine learning techniques should pave the way for the discovery of novel biomarkers and therapeutic targets for personalized therapy at a much lower cost.

## Author contributions

**Songjie Han:** methodology, investigation, writing – review & editing, visualization; **Herong Cui:** data curation, writing – review & editing; **Qianqian Xu:** writing – review & editing, supervision; **Chuwei Tang:** software; **Xiaofeng Xia:** resources; **Rui Zheng:** resources; **Yang Sun:** formal analysis; **Hongcai Shang:** conceptualization, writing – review & editing, supervision, project administration, funding acquisition. All authors read and approved the final manuscript.

## Conflict of interests

The authors have declared no competing interests.

## Funding

This work was supported by the 10.13039/501100001809National Natural Science Foundation of China (No. 82204943), the 10.13039/501100002858China Postdoctoral Science Foundation (No. 2022M710471), the New Teacher Program of Beijing University of Chinese Medicine (2023-JYB-XJSJJ034) and the 2023 Graduate independent research Project of Beijing University of Chinese Medicine (90011461220378) awarded.
