# Supplementary material for: Single-cell spatial transcriptomics in cardiovascular development, disease, and medicine
Source: Genes Dis. 2023 Nov 14;11(6):101163. doi: 10.1016/j.gendis.2023.101163 (PMC11367031; doi:10.1016/j.gendis.2023.101163)
Supplement: Multimedia component 1 [file mmc1.docx]

**Table S1 scRNA-seq studies of cardiovascular cell atlas**

| **Organ system** | **Species / genotype** | **Methods** | **Number of cells for analysis** | **DOI** |
| --- | --- | --- | --- | --- |
| Fibroblast | Mice | scRNA-seq | 44949 | 10.1161/CIRCRESAHA.119.315491. |
| Fibroblast and vascular parietal cells | Mice | scRNA-seq | 6158 | 10.1038/s41467-020-17740-1 |
| Non-myocardial cells | Mice | scRNA-seq | ＞12000 | 10.1016/j.celrep.2017.12.072 |
| Macrophages | Mice | scRNA-seq | 76 AV nodes | 10.1016/j.cell.2017.03.050 |
| Endothelial cells | Mice | scRNA-seq | ＞32000 | 10.1016/j.cell.2020.01.015 |
| Embryonic stem cells | Human | scRNA-seq | 12493 | 10.1016/j.stemcr.2019.01.022 |
| Embryonic heart | Zebrafish | scRNA-seq | 34683 | 10.1021/acs.est.0c02428 |
| Embryonic cell | Mice | scRNA-seq | 2465 | 10.1038/s41598-018-30551-1 |
| Embryonic cells | Mice | scRNA-seq | ＞9000 | 10.3791/60647 |
| Embryonic heart | Human | scRNA-seq | 4000 | 10.1016/j.celrep.2019.01.079 |
| Embryonic heart | Human | scRNA-seq & ST | 3717 | 10.1016/j.bbrc.2016.04.139 |
| Embryonic heart | Mice | scRNA-seq | ＞10，000 | 10.1016/j.celrep.2016.12.040 |
| Embryonic heart | Rats | scRNA-seq | >100，000 | 10.1038/nature18633 |
| Embryonic heart | Mice | scRNA-seq | 1205 | 10.1242/dev.173476 |
| Embryonic heart | Mice | scRNA-seq | ＞10，000 | 10.1038/s41586-019-1414-x |
| Embryonic heart | Mice | scRNA-seq | ＞36，000 | 10.1242/dev.180398 |
| Embryonic heart | Mice | scRNA-seq | ＞75，000 | 10.1242/dev.173476 |
| Embryonic heart | Mice | scRNA-seq | 2233 | 10.1186/s13059-018-1416-2 |
| Embryonic heart | Mice | scRNA-seq | 1916 | 10.1016/j.devcel.2016.10.001 |
| Embryonic heart | Mice | scRNA-seq | >1200 | 10.1126/sciadv.abf7910 |
| Embryonic heart | Mice | scRNA-seq | 1044 | 10.1016/j.celrep.2020.107739 |
| Pericardial cells | NA | scRNA-seq | 7181 | 10.1172/jci.insight.139377 |
| Myocardial B cells | Mice | scRNA-seq | 5000 | 10.1007/s00395-019-0765-7 |
| Myocardial cells | Mice | scRNA-seq | 213 | 10.1016/j.vascn.2020.106915 |
| Myocardial cells | Human | scRNA-seq | 1421 | 10.1016/j.yjmcc.2021.09.002 |
| Myocardial cells | Mice | scATAC-seq | NA | 10.1161/CIRCRESAHA.119.314908 |
| Myocardial cells | Human | scRNA-seq | NA | 10.1007/s00395-019-0744-z |
| Myocardial cells | Human | scRNA-seq | 586 | 10.7554/eLife.50163 |
| Myocardial cells | Zebrafish | scRNA-seq | 768 | 10.3389/fcell.2021.787684 |
| Myocardial cells | Human | scRNA-seq | 7775 | 10.1161/CIRCULATIONAHA.120.050635 |
| Myocardial cells | Mice, Rats | scRNA-seq | NA | 10.1038/s41467-018-07333-4. |
| Myocardial cells | Human | scRNA-seq | 10376 | 10.1089/scd.2019.0030 |
| Myocardial cells | Human | scRNA-seq | 85 | 10.1371/journal.pgen.1009666 |
| Myocardial cells | Human | scRNA-seq | 1762 | 10.1038/nature24454 |
| Myocardial cells | Mice | scRNA-seq | ＞2000 | 10.3389/fimmu.2022.973211 |
| Myocardial cells | Human | scRNA-seq & ST | 40868 | 10.1111/bph.15755 |
| Cardiac endothelial cells | Mice | scRNA-seq | NA | 10.1073/pnas.1702295114 |
| Cardiac endothelial cells | Mice | scRNA-seq | NA | 10.1161/CIRCULATIONAHA.119.041433 |
| Cardiac endothelial cells | Mice | scRNA-seq | 100000 | 10.1016/j.yjmcc.2021.09.009 |
| Epicardial cells | NA | scRNA-seq | 12608 | 10.1161/CIRCULATIONAHA.121.055468 |
| Epicardial cells | Zebrafish | scRNA-seq | NA | 10.1016/j.yjmcc.2021.09.011 |
| Heart | Mice | scRNA-seq & ST | NA | 10.3390/jcdd9010001 |
| Heart | Mice | ST | 5000 | 10.3390/cells9051144 |
| Heart | Mice | scRNA-seq | 100605 | 10.1038/s41467-021-21892-z |
| Heart | Chicken embryos | scRNA-seq & ST | 22000 | 10.3389/fcell.2021.715093 |
| Cardiac endothelial cells and myocardial cells | Mice | scRNA-seq | NA | 10.1016/j.bbadis.2020.165917 |
| Epicardial cells | Human | scRNA-seq | NA | 10.1242/dev.185652 |
| Heart cells | Zebrafish | scRNA-seq | 2885 | 10.1152/ajpheart.00056.2021 |
| Heart cells | Mice | scRNA-seq | 1000-5800 | 10.1161/CIRCULATIONAHA.119.045401 |
| Heart cells | Human | scRNA-seq | 287269 | 10.1038/s41467-018-02891-z |
| Heart progenitor cells | Mice | scRNA-seq | 122 | 10.1038/s41467-018-07307-6 |
| Heart progenitor cells | Mice | scRNA-seq | 421 | 10.1371/journal.pbio.3001200 |
| Heart progenitor cells | Human | scRNA-seq | 1024 | 10.1371/journal.pbio.3001200 |
| Heart progenitor cells | Mice | scRNA-seq | 3,494 | 10.1186/s12915-019-0709-6 |
| Heart progenitor cells | Human | scRNA-seq | 6879 | 10.1242/dev.200189 |
| Pericytes | Zebrafish | scRNA-seq | 12865 | 10.1038/s41467-020-20448-x |
