# Supplementary material for: Single-cell spatial transcriptomics in cardiovascular development, disease, and medicine
Source: Genes Dis. 2023 Nov 14;11(6):101163. doi: 10.1016/j.gendis.2023.101163 (PMC11367031; doi:10.1016/j.gendis.2023.101163)
Supplement: Multimedia component 2 [file mmc2.docx]

**Table S2 scRNA-seq studies of specific areas of the heart**

| **Organ system** | **Species / genotype** | **Methods** | **Number of cells foranalysis** | **DOI** |
| --- | --- | --- | --- | --- |
| Sinoatrial node | Mice | scRNA-seq | 77125 | 10.1242/dev.199752 |
| Atrioventricular canal | Zebrafish | scRNA-seq | 511 | 10.1038/s41586-018-0288-7 |
| Coronary vessels | Mice | scRNA-seq | 915 | 10.1093/cvr/cvac023 |
| Coronary vasculature | Human | scRNA-seq | ＞10，000 | 10.1038/s41467-021-24414-z |
| Coronary vasculature | Mice | scRNA-seq | 5841 | 10.1016/j.devcel.2020.04.005 |
| Embryonic cardiac neural crest | Chicks | scRNA-seq | 156 | 10.1161/RES.0000000000000534 |
| Extraembryonic mesoderm | Mice | scRNA-seq | NA | 10.1186/s12920-021-01011-z |
| Ventricular septum | Pig | scRNA-seq | NA | 10.1016/j.devcel.2018.03.019 |
| Epicardium | Mice | scRNA-seq | 18757 | 10.1242/dev.173047 |
| Cardiac valve cells | Mice | scRNA-seq | 594 | 10.1161/CIRCRESAHA.118.314578 |
| Cardiac conduction system | Mice | scRNA-seq | ＞22000 | 10.1038/s41586-022-04760-8 |
| Cardiac parasympathetic | Mice | scRNA-seq | NA | 10.7554/eLife.70246 |
| Cardiac coronary vessels | Mice | scRNA-seq | 2339 | 10.1093/jmcb |
| Cardiac mesenchymal cells | Zebrafish | scRNA-seq | 18739 | 10.1016/j.celrep.2019.06.092 |
| Cardiac outflow tract (OFT) | Mice | scRNA-seq | 55611 | 10.1016/j.devcel.2020.01.023 |
| Cardiac-epicardium | Zebrafish | scRNA-seq | NA | 10.1161/ATVBAHA.119.312732 |
| Perivascular adipose tissue | Mice | scRNA-seq | 12158 | 10.1093/nsr/nwaa038 |
| Aorta | Mice | scRNA-seq | 216 612 | 10.1161/ATVBAHA.121.316883 |
| Aorta | Mice | scRNA-seq | 28014 | 10.1161/CIRCULATIONAHA.118.038362 |
| Aorta | Mice | scRNA-seq | > 10000 | 10.1038/s41467-018-04893-3 |
| Aorta | Mice | scRNA-seq | 542 | 10.1038/s12276-021-00671-2 |
| Aorta | Mice | scRNA-seq | 24001 | 10.1161/ATVBAHA.120.314789 |
| Aortic valve | Human | scRNA-seq | 34632 | 10.3390/cells11172711 |
| Great saphenous vein | Human | scRNA-seq | 18957 | 10.3389/fimmu.2021.763647 |
