# Supplementary material for: Single-cell spatial transcriptomics in cardiovascular development, disease, and medicine
Source: Genes Dis. 2023 Nov 14;11(6):101163. doi: 10.1016/j.gendis.2023.101163 (PMC11367031; doi:10.1016/j.gendis.2023.101163)
Supplement: Multimedia component 3 [file mmc3.docx]

**Table S3 Single-cell spatial transcriptomics in heart failure**

| **Species/genotype** | **Organ system** | **Method** | **Number of cells** | **Findings2** | **DOI** |
| --- | --- | --- | --- | --- | --- |
| Mice & Human | CMs | scRNA-seq | 280 | Cardiac dopamine D1 receptor may be a therapeutic target for the prevention of ventricular arrhythmias associated with heart failure | 10.1038/s41467-020-18128-x. |
| Mice | CMs | scRNA-seq | 482 | Nppa expression level may be a useful diagnostic marker for heart failure. | 10.1038/s41598-021-86821-y. |
| Mice | CMs | scRNA-seq、scATAC-seq、bulk ATAC-seq and miRNA-seq | 23033 | Five major cell types were identified by scRNA-seq analysis | 10.7150/thno.68232. |
| Pig | CMs | scRNA-seq | NA | Anti-mir-21 drugs can effectively inhibit cell remodeling | 10.1016/j.jacc.2020.02.041. |
| Human | CMs & Non-CMs | scRNA-seq | 13986 | ACE2 is present in both CM and non-CM, whereas the number of ACE2-positive (ACE2+) CM in these CM and ACE2 gene expression are significantly increased in failing hearts. | 10.3389/fcvm.2021.628885. |
| Human | CMs,ECs, fibroblasts | scRNA-seq | NA | Abnormal myosin subunits were found in CM in HF, including a decrease in myosin heavy chain 6, myosin light chain 7, and an increase in myosin heavy chain 7. | 10.3390/biomedicines10020402. |
| Mice | Fibroblasts | scRNA-seq | 77602 | A specific fibroblast subset present in heart failure homeostasis, acquires Thbs4 expression and expands after injury to drive cardiac fibrosis, and identifies the transcription factor TEAD1 as a regulator of fibroblast activation. | 10.1038/s41467-022-30682-0. |
| Mice | Fibroblasts | scRNA-seq & ST | 1783 | Role of cardiac fibroblasts in regulating cardiomyocyte homeostasis and cardiac fibrosis through the Htra3-TGF-β-IGFBP7 pathway | 10.1038/s41467-022-30630-y. |
| Human | Immune cell | scRNA-seq | 181712 | Circulating cells derived from patients with heart failure have an altered phenotype, and increased FABP5 and Wnt signaling features may contribute to enhanced monocyte activation | 10.1093/cvr/cvaa101 |
| Human | Monocytes | scRNA-seq | 77278 | There is a high inflammatory transcriptome in peripheral blood monocytes and T cells of heart failure patients with DNMT3A mutation, which may be involved in the aggravation of chronic heart failure. | 10.1161/CIRCRESAHA.120.317104. |
| Mice | Non-fibroblasts | scRNA-seq | 26077 | Histone deacetylation (HDAC) inhibitors and HSP inhibitors have been identified as potential new drugs against heart failure. | 10.1016/j.ygeno.2022.110322. |
| Mice | RBC-Evs | scRNA-seq | 42497 | The complex cellular network of RBC-EV-mediated intercellular communication and its functional role in ischemic heart failure were revealed | 10.26508/lsa.202101048. |
| Mice | T cells | scRNA-seq | 20000 | CD8 +T cells regulate the transformation of cardiac resident and infiltrating macrophages into cardioprotective macrophages | 10.3389/fimmu.2021.763647. |
