# Supplementary material for: Single-cell spatial transcriptomics in cardiovascular development, disease, and medicine
Source: Genes Dis. 2023 Nov 14;11(6):101163. doi: 10.1016/j.gendis.2023.101163 (PMC11367031; doi:10.1016/j.gendis.2023.101163)
Supplement: Multimedia component 4 [file mmc4.docx]

**Table S4 Single-cell spatial transcriptomics in atherosclerosis**

| **Species/genotype** | **Organ system** | **Method** | **Number of cells** | **Findings2** | **DOI** |
| --- | --- | --- | --- | --- | --- |
| Human | Vascular cell | scRNA-seq | 3282 | Cardiovascular disease susceptibility genes are abundantly expressed in macrophages, endothelial cells and smooth muscle cells of lesions | 10.1161/CIRCRESAHA.120.316770. |
| Mice | Macrophage | scRNA-seq | ＞200 | Jak2VF macrophage proliferation and increased glycolytic metabolism lead to DNA replication stress and AIM2 inflammasome activation, which aggravates atherosclerosis. Therapies targeting interleukin-1β or specific inflammasomes substantially reduce cardiovascular risk. | 10.1038/s41586-021-03341-5. |
| Mice & Human | Smooth muscle cell | scRNA-seq | 15896 | The integration of cell-specific fate maps, single-cell genomics, and human genetics has led to a new understanding of the complexity of SMC biology. | 10.1161/CIRCULATIONAHA.120.048378. |
| Mice | Aortic leukocytes | scRNA-seq | 1138 | Definition of leukocyte diversity Fine-grained analysis of aortic leukocyte subsets reveals novel immune mechanisms and cell type-specific pathways. | 10.1161/CIRCRESAHA.117.312513. |
| Mice | CD45 | scRNA-seq | NA | Treg plays an important role in the resolution of atherosclerotic cardiovascular disease. | 10.1161/CIRCRESAHA.119.316461. |
| Mice | ECs | scRNA-seq | 520133 | Phenotypic and metabolic heterogeneity of endothelial cells in diabetes-related atherogenesis has been described at the single-cell level. | 10.3389/fcell.2021.689469. |
| Mice | ECs | scRNA-seq | 991 | JAG1-NOTCH4 sensing of perturbed flow enhances susceptibility to atherosclerosis by modulating EC heterogeneity. | 10.1126/sciadv.abo7958. |
| Human | CD^+^4 T cells | scRNA-seq | 41782 | CAD and DM were clearly reflected in the PBMC transcriptome and were significantly different between females and males and between DM and non-DM. | 10.3390/ijms23179875. |
| Human | Arteria carotis | scRNA-seq | 51981 | TNFa signaling drives inflammation in PA ECs and VSMCs. | 10.1038/s42003-022-04056-7. |
| Human | Monocytes | scRNA-seq | 239 | Overexpression of CCXCL3, GK, FPR1, and LST1 are advanced identification and intervention factors for unstable plaques. | 10.1155/2022/3972272. |
| Mice | Smooth muscle cell | scRNA-seq | ＞80000 | There is a hierarchical organization of regulatory modules that fine-tune cell states working together. | 10.3389/fgene.2022.900358. |
| Mice | Smooth muscle cell | scRNA-seq | 10138 | The specific spatio-temporal characteristics of the RGS5high VSMC subset suggest that this subset is involved in vascular homeostasis. | 10.1371/journal.pone.0265132. |
| Mice | NA | scRNA-seq | 20309 | Aortic B cells predominate in atherosclerosis. | 10.1007/s13238-021-00904-0. |
| Human | Smooth muscle cell | scRNA-seq | 6191 | Loci associated with CAD revealed significant levels of association mainly in the plaque SMC and EC populations. | 10.1093/ehjopen/oeab043. |
| Mice | Smooth muscle cell | scRNA-seq | 680 | GDF10-mediated VSMC osteogenic switch, with a likely detrimental role in atherosclerotic plaque stability. | 10.3390/ijms23031796. |
| Mice | Macrophage | scRNA-seq | 1059 | LYVE-1 res-like macrophages, via the secretion of CCL24, promote the transdifferentiation of VSMC to osteogenic-like cells. | 10.3390/cells11030411. |
| Mice | Macrophage | scRNA-seq | 11  clusters of WBC | Intimal nonfoamy macrophages are the major population expressing IL(interleukin)-1β and many other inflammatory transcripts in the atherosclerotic aorta. | 10.1161/CIRCRESAHA.118.312804. |
| Mice | Smooth muscle cell | scRNA-seq | NA | P16 and p16-driven reporter genes have significant limitations in identifying and removing senescent cells in atherosclerosis. | 10.1093/cvr/cvab208. |
| Mice | Smooth muscle cell | scRNA-seq | NA | Ampa-type glutamate receptors are expressed in VSMCS and have been implicated in phenotypic modulation. | 10.3389/fcvm.2021.655869. |
| Mice | Smooth muscle cell | scRNA-seq | NA | Cholesterol exposure activated all 3 pathways of the UPR and induced phenotypic switching primarily through the Perk-eIF2α-Atf4 arm of ER stress. | 10.1161/ATVBAHA.120.315164. |
