# Supplementary material for: Single-cell spatial transcriptomics in cardiovascular development, disease, and medicine
Source: Genes Dis. 2023 Nov 14;11(6):101163. doi: 10.1016/j.gendis.2023.101163 (PMC11367031; doi:10.1016/j.gendis.2023.101163)
Supplement: Multimedia component 5 [file mmc5.docx]

**Table S5 Single-cell spatial transcriptomics in myocardial infarction**

| **Species/genotype** | **Organ system** | **Method** | **Number of cells** | **Findings2** | **DOI** |
| --- | --- | --- | --- | --- | --- |
| Human | Cell | scRNA-seq | 4933 | LAUR(urokinase plasminogen activator receptor) is involved in the expression of genes involved in cellular response to hypoxia. | 10.3934/mbe.2021386. |
| Mice | Fibroblasts | scRNA-seq | 29176 | CTHRC1 is a novel regulator in the process of scar healing. | 10.1161/CIRCULATIONAHA.119.044557. |
| Mice | Neonatal heart | scRNA-seq | 17000 | This study constructed a comprehensive database documenting single-cell gene expression and open chromatin landscape at different stages after regenerative and nonregenerative heart injury. | 10.1016/j.celrep.2020.108472. |
| Mice | Neutrophile granulocyte | scRNA-seq | 1723 | Provides a high-resolution, dynamic blood and cardiac neutrophil census of post-MI heterogeneity. | 10.1161/CIRCRESAHA.120.317200. |
| Mice | Cells | scRNA-seq | ＞30000 | The nonlinear dynamics of myeloid and fibroblast lineages following cardiac injury are highlighted, providing entry points for in-depth analyses of cardiac homeostasis, inflammation, fibrosis, repair, and regeneration. | 10.7554/eLife.43882. |
| Rats | Endotheliocyte | scRNA-seq | 7150 | A single-cell gene expression profile of heart-specific resident endothelial cells is presented, as well as the transcriptional hierarchy of endogenous vascular repair after MI. | 10.1093/eurheartj/ehz305. |
| Human | Heart | ST | 191795 | A complete molecular map of human myocardial infarction is provided. | 10.1038/s41586-022-05060-x. |
| Mice | Immune cell | scRNA-seq & ST | 533 | The immune response driven by mi was understood and the regulators of Trem2 signaling pathway were identified. | 10.1038/s41467-022-32284-2. |
| Mice | B cell | scRNA-seq | 6588 | Polyclonal B cells without antigen specificity readily infiltrate the heart after MI via the CXCL13-CXCR5 axis and promote local TGF-β1 production. | doi: 10.1093/cvr/cvab181. |
| Human | CXCR7 | scRNA-seq | 834 | CXCR7 may be a new therapeutic target for myocardial infarction. | 10.1038/s41598-021-83022-5. |
| Mice | Monocytes /Macrophage | scRNA-seq | 6503 | Studies have provided a set of monocyte-associated biomarkers and potential therapeutic targets for HF after AMI. | 10.1186/s12920-021-00890-6. |
| Human | Pericyte | scRNA-seq & ST | 65054 | Specific pericyte markers in lung, heart, kidney, and bladder were identified for the first time, and differentially expressed genes and functional relationships among parietal cells were revealed. | 10.3389/fcvm.2022.876591. |
| Mice | Neutrophile granulocyte | scRNA-seq | ＞28000 | The study provided a spatial and temporal map of neutrophil specialization in response to MI and revealed dynamic pro-inflammatory cardiac Ly6GSigF (MycNFκB) neutrophils that had been overlooked due to negative selection. | 10.1161/JAHA.120.019019. |
| Mice | RBC-EVs | scRNA-seq | 42497 | The complex cellular network of erythrocyte-EV-mediated intercellular communication in ischemic heart failure was revealed | 10.26508/lsa.202101048. |
| Human | White blood cell | scRNA-seq | 2224 | PRKAR1A and SDCBP can be used as novel biomarkers for early diagnosis of AMI after HF | 10.3389/fimmu.2022.878876. |
| Mice | Neutrophile granulocyte | scRNA-seq | 2757 | Neutrophil-derived alarmin (S100A8/A9) plays a major role in determining the nature of the subsequent inflammatory response after myocardial injury. | 10.1161/CIRCULATIONAHA.119.043833. |
| Human | Cells | scRNA-seq | 1084 | The study revealed the immune characteristics of myocardial infarction at the single-cell level and identified 7 prognostic markers. | 10.1155/2022/6534126. |
| Rat/Pig | CMs | scRNA-seq | 7000 | Studies provide insights into the process of mandatory cardiomyocyte proliferation and minimize the oncogenic potential of cyclin by using a novel transient and cardiomyocyte-specific viral construct. | 10.1161/CIRCULATIONAHA.121.057641. |
| Mice | Mesenchymal cell | scRNA-seq | 36847 | The early increase in activated myofibroblasts, enhanced collagen deposition, and persistent acute phase response in the hearts of 129S1/SvImJ mice define a critical time window for pathological remodeling that is predictive of disease outcome. | 10.1016/j.celrep.2020.02.008. |
| Mice | ECs | scRNA-seq | 7150 | A single-cell gene expression profile of heart-specific resident endothelial cells is presented, as well as the transcriptional hierarchy of endogenous vascular repair after MI. | 10.1093/eurheartj/ehz305. |
| Mice | CVPs | scRNA-seq | NA | Single-cell RNA sequencing of CVPS derived from human ES cell lines supported reproducibility and identified three major progenitor cell subsets. | 10.1016/j.celrep.2019.02.083. |
| Mice | Fibroblasts/Stromal cells | scRNA-seq | 13796 | Important epicardial properties of wound healing/regeneration after MI are attributed to specific cell populations. | 10.7554/eLife.65921. |
| Mice | ECs | scRNA-seq | 35312 | Transient mesenchymal activation of endothelial cells was demonstrated. | 10.1038/s41467-021-20905-1. |
| Mice | Tregs | scRNA-seq | NA | Sparc, which is highly expressed by cardiac Tregs, is a key factor in protecting the heart from MI by increasing collagen content and promoting the maturation of the infarct zone. | 10.1161/CIRCULATIONAHA.120.046789. |
