# Supplementary material for: Single-cell spatial transcriptomics in cardiovascular development, disease, and medicine
Source: Genes Dis. 2023 Nov 14;11(6):101163. doi: 10.1016/j.gendis.2023.101163 (PMC11367031; doi:10.1016/j.gendis.2023.101163)
Supplement: Multimedia component 6 [file mmc6.docx]

**Table S6 Single-cell spatial transcriptomics in other diseases**

| **Species/genotype** | **Organ system** |  | **Method** | **Number of cells** | **Findings2** | **DOI** |
| --- | --- | --- | --- | --- | --- | --- |
| Human | Coronary artery endothelium |  | ST | NA | Alterations in myocardial programming during COVID-19 have been linked to capillary endothelial cells, with endotheliitis and reduced endothelial activation being key drivers of cardiovascular events during COVID-19. | 10.1101/2022.09.25.509426. |
| Mice | Cells |  | scRNA-seq | 16000 | M2 cells, T cells and fibroblasts may be involved in the pathogenesis of viral myocarditis. | 10.1016/j.isci.2022.103865. |
| Human | PBMC |  | scRNA-seq | 12000 | High expression of proinflammatory mediators and immunoglobulin receptors, low expression of MHC class II genes, and oligoclonal expansion of B-cell and T-cell receptors after treatment in acute KD. | 10.1038/s41467-021-25771-5. |
| Human | Hematopoietic cell |  | scRNA-seq | 19197 | The functions of up-regulated genes in B cells and four kinds of T cells in children with Kawasaki disease were mainly cell activation, lymphocyte activation and the regulation of immune system processes. | 10.1186/s12887-021-02754-5. |
| Human | IPSC-Cms |  | scRNA-seq | 25619 | An integrated approach that can elucidate multiple possible mechanisms that trigger disease is demonstrated. | 10.1007/s10439-021-02850-8. |
| Human | Vascular cell |  | scRNA-seq | 6487 | Enhanced understanding of the underlying mechanisms of ASD at the cellular and molecular levels and highlighted potential targets for ASD treatment. | 10.1111/jcmm.16914. |
| Mice | Immune cell |  | scRNA-seq | 13 clusters | There were differences in immune infiltration between HCM patients and normal subjects. | 10.1155/2022/7153491. |
| Rats | Myocardial cell |  | scRNA-seq & ST | 29004 | The data demonstrate the utility of using scRNA-seq for human hypertrophic hearts, highlighting the heterogeneity of CM. | 10.1016/j.celrep.2022.110809. |
| Human | IPSC-Cms |  | scRNA-seq | 1000 | Hipsc-derived engineered tissues provide a unique approach to study primary cardiac abnormalities and reveal the pathogenic mechanisms of sporadic congenital heart disease. | 10.1161/JAHA.120.016528. |
| Human/Rats | Smooth muscle cell |  | scRNA-seq | NA | BAF60c plays an important role in maintaining VSMC homeostasis and has great potential in the prevention and treatment of AAA. | 10.1172/JCI158309. |
| Human | Stromal valve interstitial cells |  | scRNA-seq | 12776 | Aortic valve cells are heterogeneous, and mVICs secrete MDK to prevent VICs calcification. MDK may provide a potential therapeutic strategy as a novel inhibitor of CAVD. | 10.3389/fcell.2021.794058. |
| Mice | Vascular cell |  | scRNA-seq | 5000 | Aortic vascular cells associated with hypertension have a favorable transcriptional landscape, with significant changes in cell composition and intercellular communication during the progression of hypertension. | 10.1038/s12276-021-00704-w. |
| Mice/Human | T cells |  | scRNA-seq | 53346 | Klf10 or IL-9 may represent novel therapeutic targets for the treatment of vascular or fibrotic diseases. | 10.1161/CIRCRESAHA.121.320420. |
| Rats | Vessel |  | scRNA-seq | 28977 | Transient ACE inhibition alters cardiac fibroblast subsets and degree of activation, resulting in a reduced overall fibrotic phenotype. | 10.1161/HYPERTENSIONAHA.120.16352. |
| Mice | Aortic valve |  | scRNA-seq | 6574 | Activation of PPARγ protected the anti-inflammatory effects of the aortic valve. | 10.1038/s41467-022-33202-2. |
| Mice | CD45 + |  | scRNA-seq | 46040 | HIF1A can be used as a potential target for attenuating AR. | 10.1007/s00395-021-00904-5. |
| Mice | Macrophage  /ECs |  | scRNA-seq | 21 | The cellular heterogeneity within the graft was comprehensively analyzed and the specific macrophage and endothelial cell populations that mediate rejection were described, providing a potential predictive biomarker for rejection. | 10.3389/fimmu.2022.832573. |
| Mice | CD45 + |  | scRNA-seq | 18678 | CXCR3 pathway can be used as a potential therapeutic target for transplantation rejection. | 10.7150/thno.75543. |
| Human | CDCs |  | scRNA-seq | 2815 | CDCs are mitochondria-rich cells that exhibit highly proliferative, secretory, and immunomodulatory properties. | 10.1007/s00395-022-00913-y. |
| Human | Cells |  | scRNA-seq | 5000 | Potential impairment of TGF-β signaling and lack of stimulation of SMC differentiation. | 10.3390/genes13010095. |
| Mice | Smooth muscle cell |  | scRNA-seq | > 9800 | Single-cell RNA sequencing identified TGF-β signaling and Klf4 overexpression as potential upstream drivers of SMC regulation. | 10.1161/ATVBAHA.120.314670. |
| Human | Intima of the lung |  | scRNA-seq | 27140 | The order of single cells along developmental trajectories in CTEPH and the close communication between different cell types in the pathogenesis of CTEPH were revealed. | 10.1161/HYPERTENSIONAHA.121.18105. |
| Mice | Fibroblasts |  | scRNA-seq | 16874 | Single-cell sequencing of healthy and diseased adult hearts has allowed us to investigate transcriptomic differences between cardiac cells, as well as cell type-specific gene expression changes during cardiac disease. | 10.1161/CIRCULATIONAHA.117.030742. |
| Mice | Myocardial cell |  | scRNA-seq | NA | Activation of cardiomyocyte proliferation and a supportive environment are key to the occurrence of regeneration. | 10.1093/cvr/cvac155. |
| Human/Mice | Heart |  | scRNA-seq & ST | NA | SOX9 is a potential therapeutic target for cardiac fibrosis. | 10.1161/CIRCULATIONAHA.117.027832. |
| Pig | Epicardial adipose tissue stem cells |  | scRNA-seq | 25521 | The unique phenotype of the heterogeneous EATDS population opens important translational opportunities for myocardial regeneration and cardiac management. | 10.1007/s12015-021-10273-0. |
| Mice | ECs |  | scRNA-seq | 472930 | Defining cardiac aging in the context of the endothelial transcriptome to understand the cellular factors that disrupt each organ system during aging. | 10.1152/physiolgenomics.00037.2021. |
| Mice | ECs |  | scRNA-seq | 1441 | Studies have revealed the reprogramming of the transcriptome in cardiovascular endothelial cells of aging mice, which has deepened the understanding of the molecular mechanisms of cardiovascular aging. | 10.3389/fcvm.2022.900978. |
| Mice | Smooth muscle cell |  | scRNA-seq | 607 | Disruption of endothelium-mediated nitric oxide (NO) signaling in no3 -/- mice leads to aortic dilatation and dissection, which is the result of inhibition of VSMCs elastic fiber formation in the ascending aorta. | 10.1242/dmm.044990. |
| Human | Fetal heart |  | scRNA-seq | 157273 | Cardiac fibroblasts in HLHS were enriched in the low Hippo and high YAP cell state characteristics of activated cardiac fibroblasts. | 10.1038/s41586-022-04989-3. |
| Human | Cells |  | scRNA-seq | 17747 | Increased and heterogeneous interferon responses in different cell types of CHB hearts, enriched maternal body transcripts in stromal cells may contribute to extracellular matrix deposition and subsequent fibrosis. | 10.1093/cvr/cvz257. |
| Rats | Immune cells |  | scRNA-seq | 60981 | Heart-resident macrophages are a heterogeneous population of immune cells that play a key role in stimulating angiogenesis and inhibiting fibrosis in response to cardiac pressure overload. | 10.1161/CIRCRESAHA.121.319737. |
| Mice | CD4/CD8 T cells |  | scRNA-seq | NA | CD4 T cells play a unique role in regulating cardiac regeneration and repair during development. | 10.7150/thno.42943 |
| Zebra fish | Fibroblasts |  | scRNA-seq | 1400 | Prrx1b expression in EPDC limited the number of profibrotic fibroblasts and stimulated cardiomyocyte proliferation. | 10.1242/dev.198937. |
| Cells | BNT162b2 |  | scRNA-seq | 10000 | Importance of classical monocytes in the pathogenesis of myocarditis after BNT162b2 vaccination. | 10.3389/fimmu.2022.979188. |
| Mice | Mitochondria |  | scRNA-seq | 396 | Ndufs1 is downregulated in hypertrophic cardiac tissue, and Ndufs1 deficiency may lead to mitochondrial dysfunction in cardiomyocytes, which is associated with the development and progression of CH. | 10.1155/2021/5545261. |
| Mice | Cells |  | scRNA-seq | 11492 | The dynamics of all major cardiac cell types during disease progression are elucidated, with cell type crosstalk that dynamically changes during pathological cardiac hypertrophy. | 10.1161/CIRCULATIONAHA.119.043053. |
| Human | Heart |  | scRNA-seq & ST | 23772 | There are different bone marrow cell populations and different immune cell populations with different molecular characteristics in cardiac sarcoidosis, which constitute sarcomatous granuloma. | 10.1161/CIRCRESAHA.121.320449. |
| Mice | Heart/Spleen |  | scRNA-seq | 2431 | Role of Treg in neonatal cardiac regeneration. Treg can directly promote cardiomyocyte proliferation in a paracrine manner. | 10.7150/thno.32734. |
| Human | Vessel |  | scRNA-seq | 35245 | Transcriptomic features of CVM and VM cells were described at the single-cell level. | 10.1016/j.yjmcc.2021.09.004. |
| Human | Monocyte |  | scRNA-seq | 14147 | Hemodialysis can significantly lead to abnormal PI3K-Akt-mTOR, MAPK, TNF, and NF-κB pathways in CD4+ T cell subsets. | 10.3389/fimmu.2022.878226. |
| Mice | Macrophage |  | scRNA-seq | 4358 | It was demonstrated that AT residency is associated with Lyve1, Tim4, and ABCA1 expression in ATM and the acquisition of high endocytic and lysosomal capacity. | 10.1038/s41467-021-24684-7. |
| Mice | Non-myocardial cell |  | scRNA-seq | 24862 | Cardiac macrophages (CMPS) play a key role in pressure overload-induced cardiac fibrosis and dysfunction, and macrophage miR-21 is a key molecule in the pro-fibrotic effect of CMPS. | 10.1161/CIRCULATIONAHA.120.050682. |
| Mice | CD45 |  | scRNA-seq | 48913 | Subset-specific molecules that mediate immune activation may be useful targets for the diagnosis or treatment of heart failure. | 10.1161/CIRCULATIONAHA.119.041694. |
| Zebra fish | Heart |  | scRNA-seq | 12,000-12,500 | Runx1 controls the regenerative response in a variety of cardiac cell types, and targeting Runx1 represents a novel therapeutic strategy to induce endogenous cardiac repair. | 10.1242/dev.186569. |
| Mice | Heart |  | scRNA-seq | NA | Dysregulation of paracrine signaling in nonmyocardial cell types can lead to reduced cardiomyocyte proliferation and noncompaction of the myocardium. | 10.1093/eurheartj/ehab298. |
| Mice | Epicardium |  | scRNA-seq | 18000 | Cardiac fibroblasts and epithelial cells derived from the pericardium express the paracrine factors TGFβ1 and fibroblast growth factor. | 10.1161/CIRCULATIONAHA.120.052928. |
| Human | Heart |  | ST | >1000 | Tomo-Seq is able to reveal novel molecular mechanisms in human cardiomyopathy, with ZBTB11 as a novel driver of cardiomyocyte loss. | 10.1093/cvr/cvac072. |
| Human | Heart |  | scRNA-seq | 128 412 | The cellular landscapes of the 4 types of heart valves (aortic, pulmonary, mitral, and tricuspid) are different. | 10.1161/ATVBAHA.122.318314. |
| Human | Fibroblasts |  | scRNA-seq | 3199 | Specific FB subtypes have a key role in AD progression. | 10.7150/thno.66059. |
| Mice | Macrophage |  | scRNA-seq | NA | Bone marrow derived Rel mediated CD72hi macrophages play a proinflammatory role and induce cardiac injury. | 10.1093/cvr/cvab193. |
| Rats | Cells |  | scRNA-seq | ＞20000 | Systematic characterization of cell type-specific and artery type-specific transcriptomic changes during vascular remodeling in hypertension has been described. | 10.1093/cvr/cvaa164. |
| Rats | CD45 |  | scRNA-seq | 34665 | The integrated single-cell landscape of cardiac immune cells at different EAM stages was introduced. | 10.1161/CIRCULATIONAHA.119.043545. |
| Human | Myocardial cell |  | scRNA-seq | NA | The heterogeneity of HLH cardiomyocytes was elucidated based on scRNA-seq expression profiles. | 10.3389/fcell.2021.617853. |
| Human | Myocardial cell |  | scRNA-seq | 10870 | Genetic heterogeneity exists in HLHS. | 10.1161/CIRCULATIONAHA.121.056198. |
| Mice | Myocardial cell |  | scRNA-seq & ST | 7783 | The normal degree of destruction of dense cardiomyocytes may play a key role in the pathogenesis of LVNC. | 10.1161/CIRCULATIONAHA.121.056666. |
